# Navigating diagnostic challenges—distinguishing malignant melanoma and clear cell sarcoma of soft tissues: a case report and review of the literature

**DOI:** 10.1186/s13256-024-04542-y

**Published:** 2024-05-17

**Authors:** Soufiane Abdouh, Imane Boujguenna, Abdelwahed Soleh, Imad Abkari, Hanane Rais

**Affiliations:** 1 Pathology Department, Mohammed VI University Hospital Center, Marrakesh, Morocco; 2 Faculty of Medicine and Pharmacy, Ibn Zohr University, Guelmim, Morocco; 3 Orthopedic Surgery Department, Mohammed VI University Hospital Center, Marrakesh, Morocco

**Keywords:** Melanocytic-differentiated tumor, Clear cell sarcoma of soft tissues, Soft tissue neoplasms

## Abstract

**Background:**

Within the spectrum of melanocytic-differentiated tumors, the challenge faced by pathologists is discerning accurate diagnoses, with clear cell sarcoma of soft tissues standing out as a rare and aggressive neoplasm originating from the neural crest. Accounting for 1% of all soft tissue sarcomas, clear cell sarcoma of soft tissues poses diagnostic complexities, often misidentified owing to its phenotypic resemblance to malignant melanoma. This chapter delves into the intricacies of clear cell sarcoma of soft tissues, its epidemiology, characteristic manifestations, and the imperative need for a comprehensive diagnostic approach involving immunohistochemical and molecular analyses.

**Case presentation:**

A compelling case unfolds as a 25-year-old male from Morocco, initially misdiagnosed with malignant melanoma, experiences tumor recurrence on the second toe. With no history of trauma or familial neoplasia, the patient’s clinical journey is explored, emphasizing the importance of detailed clinical examinations and radiological assessments. The chapter elucidates the histopathological findings, immunohistochemical spectrum, and the correlation between clinical parameters and diagnostic inference, ultimately leading to metatarsal amputation. This clinical vignette highlights the multidimensional diagnostic process in soft tissue neoplasms, emphasizing the synergistic role of clinical, radiological, and histopathological insights.

**Conclusion:**

The diagnostic challenges inherent in melanocytic-differentiated tumors, exemplified by the rarity of soft tissue clear cell sarcoma, underscore the essential role of an integrated diagnostic approach. This concluding chapter emphasizes the perpetual collaboration required across pathology, clinical medicine, and radiology for nuanced diagnostic precision and tailored therapeutic strategies. The rarity of these soft tissue malignancies necessitates ongoing interdisciplinary engagement, ensuring the optimization of prognosis and treatment modalities through a comprehensive understanding of the diagnostic intricacies presented by clear cell sarcoma of soft tissues.

## Introduction

In the context of encountering a melanocytic differentiated tumor, pathologists are confronted with the imperative task of discerning potential diagnoses, with clear cell sarcoma of soft tissues (CCSST) being among the considerations related to diagnostic differentials. This exceedingly rare and aggressive soft tissue neoplasm originates from the neural crest [1] and comprises approximately 1% of all soft tissue sarcomas [1, 2]. Predominantly afflicting young adults and infrequently children, CCSST shares phenotypic characteristics with malignant melanoma, necessitating an investigative approach involving immunohistochemical complementation and molecular biology for precise diagnostic elucidation. Manifesting in diverse anatomical locations, CCSST predominantly manifests in the distal extremities, with its characterization first expounded by Enzinger *et al*. in 1965 [3]. Primarily involving the fascia and tendons, the malignancy’s protracted progression often culminates in delayed diagnosis, frequently at an advanced metastatic stage. Therapeutic management primarily revolves around meticulous carcinological tumor resection. This study presents an illustrative case of a 25-year-old male presenting with a tumor recurrence of CCSST initially misdiagnosed as malignant melanoma. The overarching objective is to underscore the intricacies within the diagnostic spectrum of melanocytic differentiated tumors, emphasizing the consideration of atypical differentials, particularly in young individuals presenting with soft tissue neoplasms devoid of primary cutaneous involvement.

## Case presentation

In this intriguing case study, we present the clinical scenario of a 25-year-old Moroccan man with no pertinent prior medical history who presented 2 years ago with a mass on his second toe. Initially, the lesion raised concerns of malignant melanoma owing to its varying pigmentation ranging from dark brown to black. Gross examination revealed irregular borders, asymmetry, and a raised surface with multiple nodules, along with evidence of ulceration. Following an initial biopsy, the lesion was indeed diagnosed as malignant melanoma. However, it is crucial to acknowledge that the initial misdiagnosis of malignant melanoma was likely attributable to two factors. Firstly, limitations in the biopsy analysis potentially resulted in the exclusion of the epidermis owing to artifacting. This exclusion inadvertently obscured the crucial anatomical distinction between clear cell sarcoma of soft tissue and melanoma. Secondly, the absence of immunohistochemical analysis, a vital tool for differentiating these entities, further hampered an accurate diagnosis. Following the initial diagnosis, the patient failed to adhere to the established follow-up schedule.

At 18 months later, the lesion recurred, prompting the patient to seek reevaluation at the orthopedic surgery department. Upon physical examination, a painless ulcerated tumor measuring 3 cm in diameter was observed on the right second toe. Notably, the tumor displayed fibrin and pigmented areas on its surface. Surrounding the primary tumor, several erythematous nodular lesions with a firm texture and fixed appearance were noted.

**Fig. 1:**
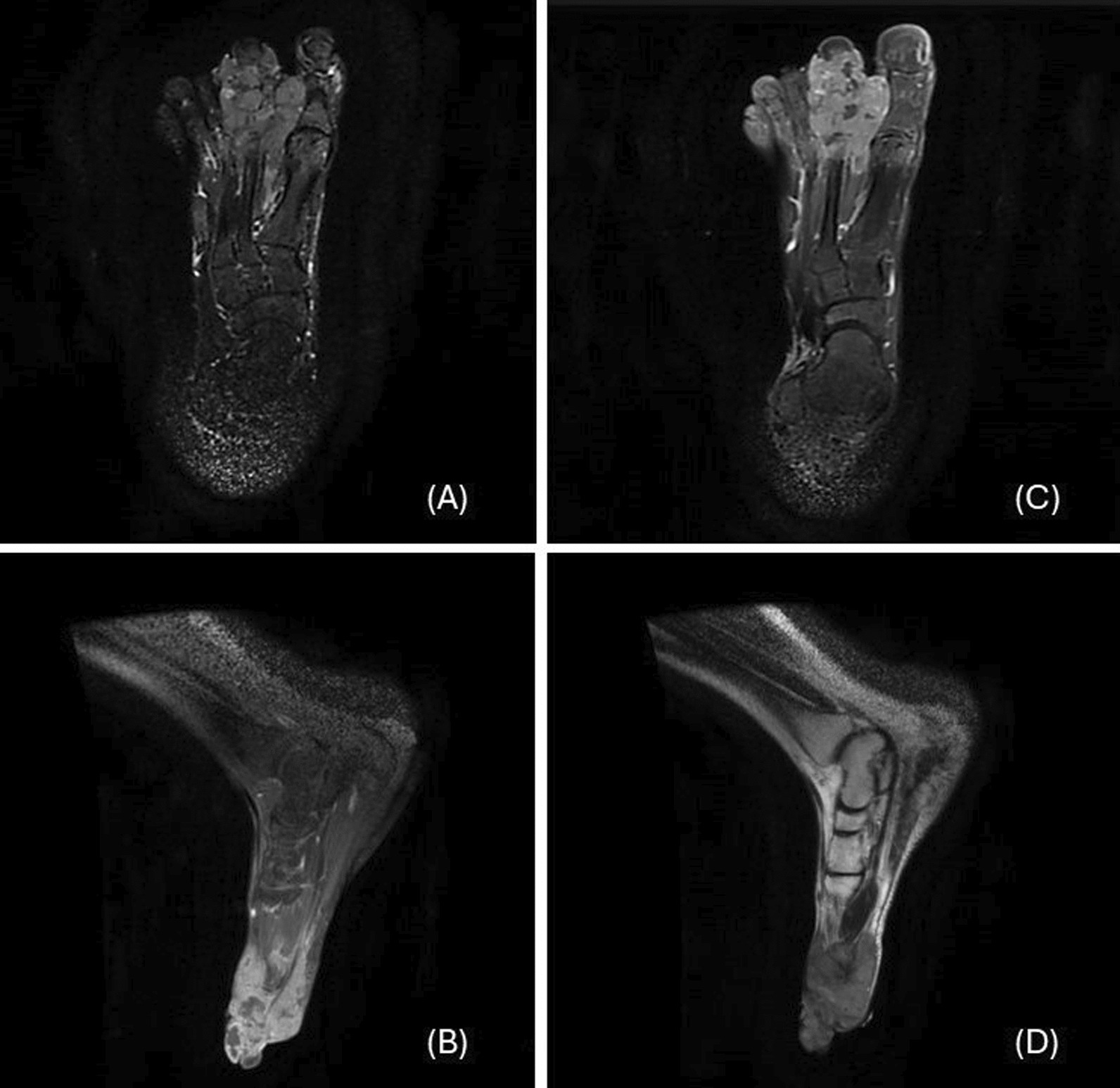
Magnetic resonance imaging assessment of the tumor process. **A.** T1-weighted axial image shows the tumor without contrast enhancement, providing detailed information about its internal structure. **B.** T1-weighted sagittal image following the administration of gadolinium contrast, it demonstrates a loss of signal intensity from the majority of the first and second phalanges, along with filling of the first and second interphalangeal joints. **C. **T2-weighted axial image. **D. **T2-weighted sagittal image. These finding offers a comprehensive visualization of the tumor’s impact on the surrounding anatomy

Radiological assessment using magnetic resonance imaging (MRI), a notable abnormality was observed in the form of a lesion resembling a giant cell tumor affecting the tendon sheaths, as depicted in Fig. 1. This imaging revealed a significant loss of tissue in the first and second phalanx, alongside a simultaneous filling of the first and second interphalangeal gaps, providing a detailed view of the tumor’s impact on the surrounding anatomy. However, despite these findings, the exact nature of the lesion remained uncertain. Potential diagnoses that were considered included giant cell tumor of the tendon sheath (GCT-TS), tenosynovial giant cell tumor (TGCT), pigmented villonodular synovitis (PVNS), synovial sarcoma, and the possibility of metastatic disease.

**Fig. 2:**
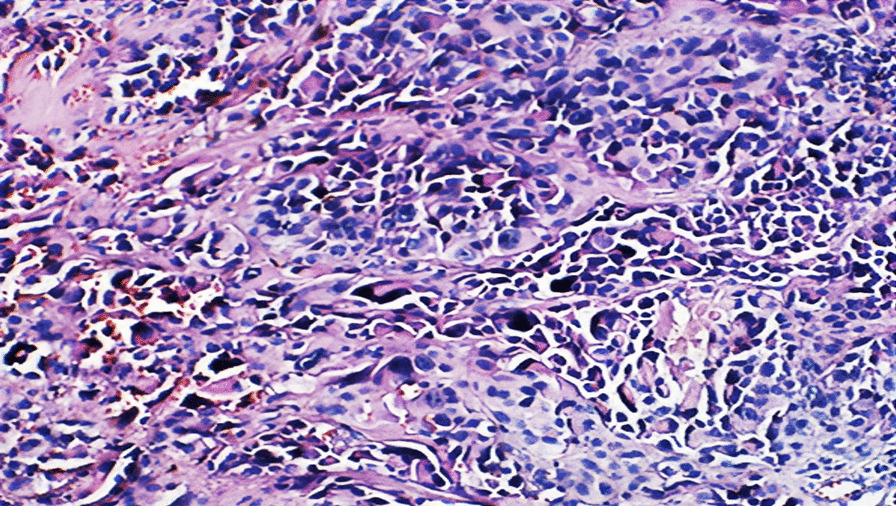
The section exhibits malignant round cell tumor proliferation separated by fibrous septa hematoxylin and eosin ×25

Subsequent excision of a nodular tumor formation, measuring 3.5 × 3 × 1.5 cm, revealed a neoplasm with firm consistency on section. Macroscopically, the lobulated and grayish skin displayed hemorrhagic foci. Microscopic examination, as depicted in Fig. 2, unveiled a tumor proliferation infiltrating the tendon, arranged in lobules demarcated by fibrous septa. The tumor cells, frequently epithelioid in appearance, exhibited small dimensions, ovoid irregularly outlined nuclei with nucleolar prominence, and pale eosinophilic cytoplasm with occasional clearing. The stroma exhibited fibroinflammatory characteristics and is devoid of epidermal involvement. Additionally, vascular emboli or necrotic foci were not observed.

**Fig. 3:**
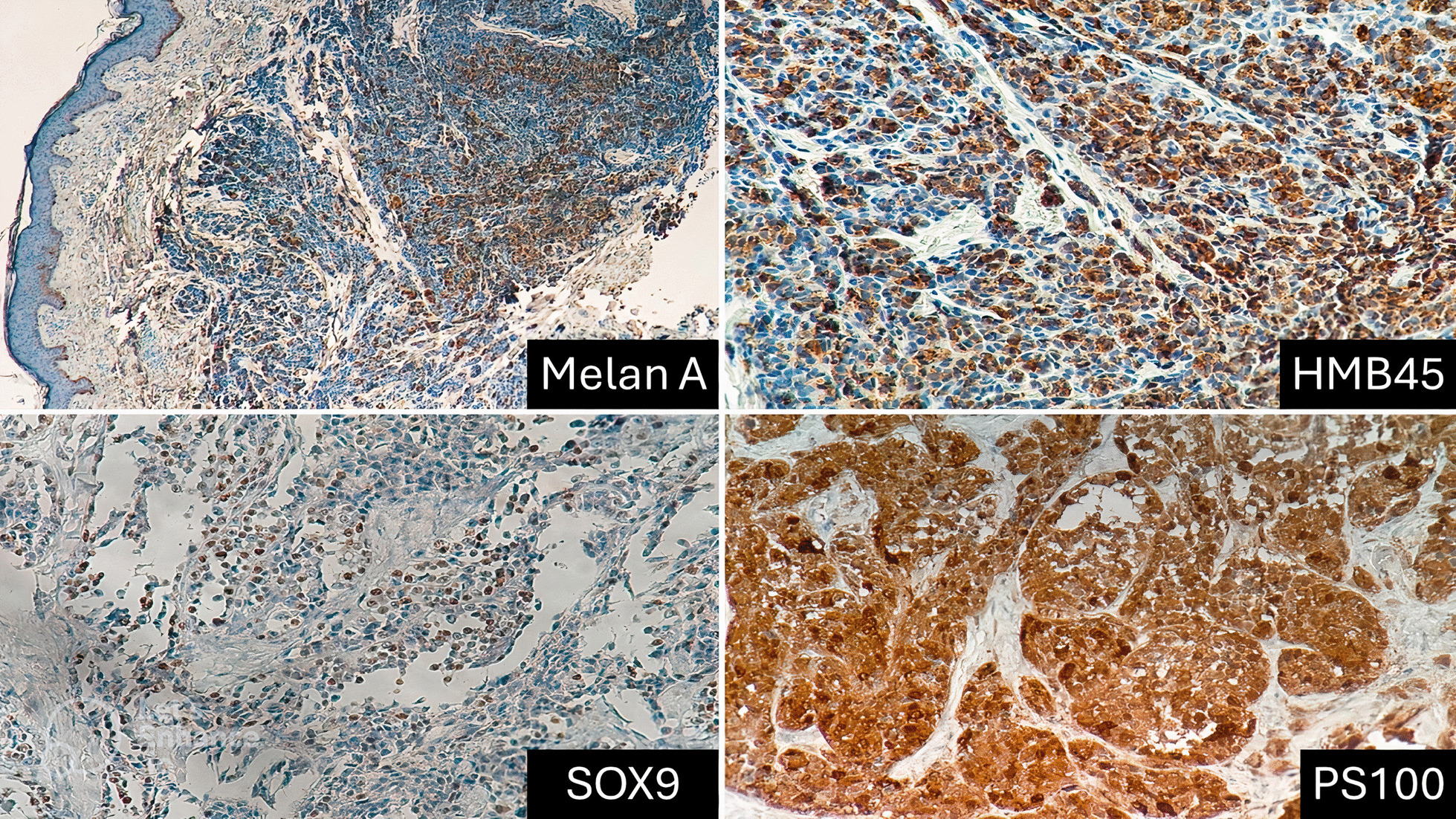
Tumor immunohistochemical profile. Immunohistochemical analysis demonstrated widespread and atypical cytoplasmic expression of HMB-45 and PS100 within tumor cells. Notably, Melan-A and SOX9 exhibited distinct staining patterns. While Melan-A showed focal cytoplasmic expression, SOX9 displayed unique nuclear extension in addition to cytoplasmic positivity. These findings offer a comprehensive characterization of the tumor’s immunophenotypic profile

Immunohistochemical analysis with antibodies against Melan-A, PS100, HMB-45, and SOX9 was performed on tissue sections (Fig. 3). All four markers demonstrated positive staining within the tumor cells.

A panel of IHC markers, including cytokeratin (CK; AE1–AE3 and CK19), FLI1, INI1, desmin, and smooth muscle actin (SMA), was employed to evaluate the origin and characteristics of the soft tissue tumor. The absence of expression in most markers, except for persistent expression of INI1 that provided valuable information in ruling out rhabdoid tumor as a potential diagnosis.

While fluorescence *in situ* hybridization (FISH) analysis for the t(12;22) (q13;q12) reciprocal translocation could further strengthen the diagnosis, its unavailability in our clinical setting did not impede the formulation of an accurate conclusion.

On the basis of the aforementioned clinical presentation, imaging findings, and particularly the immunohistochemical (IHC) analysis, a definitive diagnosis of clear cell sarcoma of soft tissue was established, effectively differentiating it from malignant melanoma.

Importantly, both physical examination and meticulous imaging studies did not reveal any evidence of metastatic spread. The tumor has been categorized as IA on the basis of the American Joint Committee on Cancer (AJCC) 2018 staging.

The patient subsequently underwent metatarsal amputation for definitive treatment. At the 12-month follow-up after metatarsal amputation, the patient remains disease-free with no signs of recurrent or metastatic disease, indicating a favorable clinical course (Table 1).

**Table 1:** Chronological summary of patient’s clinical course

T0 = initial presentation	Initial biopsy of a mass on the second toe revealed a diagnosis of malignant melanoma The patient did not adhere to the recommended follow-up schedule
T1 = 18 months later	The patient presented for consultation for recurrence of the mass The second biopsy resulted in a diagnosis of clear cell sarcoma Surgical amputation of the second toe was performed following the diagnosis
T2 = follow-up 1 year after metatarsal amputation	Disease free

## Discussion

Clear cell sarcoma of soft tissues (CCSST) is a malignant mesenchymal neoplasm primarily affecting deep soft tissues, characterized by a distinctive nested growth pattern and melanocytic differentiation. Diagnostic challenges arise owing to morphological similarities with malignant melanoma, historically leading to its classification as malignant soft tissue melanoma. Advances in molecular biology, particularly the identification of the t(12;22) (q13;q12) translocation and the resultant chimeric EWSR1/ATF1 gene fusion, have significantly enhanced diagnostic precision [4]. Although CCSST typically presents in the third and fourth decades, sporadic occurrences in the pediatric population have been documented [5].

Clinically, CCSST manifests in the lower limbs, particularly the ankle or foot of young adults, often involving tendons or fascia. Uncommon cases have been reported in the neck, gastrointestinal tract, penis, and pleural cavity [6–10].

The accurate diagnosis of clear cell sarcoma of soft tissues (CCSST) can be challenging owing to its clinical and morphological overlap with several other tumors, especially in the vicinity of tendons and aponeuroses in the extremities. Careful consideration of clinical presentation, microscopic features, and advanced techniques, such as immunohistochemistry (IHC) and fluorescence in situ hybridization (FISH), is essential for distinguishing CCSST from its mimics and ensuring optimal patient management.

Histologically, CCSST presents as a well-limited, lobulated, or multinodular tumor with dimensions ranging from 1 to 10 cm, featuring a greyish-white appearance. Characterized by monomorphic cells with a clear or slightly eosinophilic cytoplasm and basophilic, oval, vesicular nuclei, CCSST is identifiable by the presence of multinucleated tumor giant cells in about 50% of cases. Glycogen accumulation, highlighted by periodic acid–Schiff (PAS) and PAS with diastase (PAS-D) stains, imparts the characteristic clear appearance of the cells [2]. Immunohistochemistry further reveals positivity for antigens, such as HMB-45, PS100, CD99, Melan-A, NSE, and vimentin [2, 11].

Clear cell sarcoma of soft tissues (CCSST) presents a diagnostic challenge as it can closely resemble malignant melanoma (MM), resulting in diagnostic complexities. Both malignancies exhibit morphological and immunohistochemical similarities, contributing to diagnostic confusion. Traditionally, pathologists adopt a comprehensive approach encompassing various factors, including microscopic features:

Microscopic examination reveals distinctive characteristics of CCSST, typically situated deeper within the dermis, characterized by a uniform distribution of spindle cells surrounded by delicate fibrous tissue. In contrast, MM frequently originates in the epidermis, the outer layer of the skin, displaying notable nucleoli, increased mitotic activity, and migration of atypical melanocytes.

Despite the clear morphological distinctions between clear cell sarcoma and malignant melanoma, both malignancies may present overlapping immunohistochemical profiles. Immunohistochemical analysis demonstrates that CCSST expresses markers typically associated with melanocytes, including HMB-45, MiTF, S100, and Melan-A [2].

Thus, relying solely on immunohistochemistry may be insufficient to distinguish between clear cell sarcoma and melanoma owing to their shared expression of melanocytic markers, complicating the diagnostic process. This underscores the importance of thorough clinical correlation. Clear cell sarcoma of soft tissues (CCSST) typically affects individuals in the younger to middle-aged adult range and often presents with tumors localized in the extremities, while melanoma is more commonly observed in elderly individuals, often manifesting as pigmented lesions on sun-exposed skin areas. The clinical correlation can aid in guiding diagnostic considerations. Additionally, molecular investigations are essential for achieving an accurate diagnosis.

Through molecular methodologies, clear cell sarcoma of soft tissues (CCSST) is distinctly identified by a recurring chromosomal aberration, namely the t(12;22) translocation, resulting in the fusion of the EWS RNA binding protein 1 (EWSR1) gene with the ATF1 transcription factor gene. This specific rearrangement of the EWSR1/ATF1 genes serves as a defining genetic feature of CCSST, setting it apart from malignant melanoma, where such rearrangement is notably absent. Additionally, techniques, such as fluorescence *in situ* hybridization (FISH) and reverse transcriptase polymerase chain reaction (RT-PCR) play a pivotal role in differentiating synovial sarcoma from CCSST by detecting characteristic translocations or fusion transcripts, such as the t(X;18)(p11.2;q11.2) translocation or SYT/SSX fusion transcripts, respectively [2].

Other differential diagnoses include paraganglioma-like dermal melanocytic tumor (PLDM), clear cell myomelanocytic tumor (CCMT), and malignant peripheral nerve sheath tumor (MPNST), each possessing unique clinical and morphological features aiding in their differentiation from CCSST [2].

Magnetic Resonance Imaging (MRI) features of CCSST often exhibit benign characteristics, displaying modestly enhanced signal intensity on T1-weighted imaging compared with muscle [12]. Early and accurate diagnosis is paramount, as surgical excision remains the primary curative treatment. Optimal preoperative imaging, facilitated by MRI, aids surgical planning, minimizing perioperative morbidity by outlining the tumor’s relationships with adjacent tissues, including nerves and major arteries [13]. Surgical excision is presently the primary therapeutic approach for early-stage clear cell sarcoma [14–16]. Despite its application in palliative care, chemotherapy is recommended for individuals with metastatic disease, although its efficacy remains uncertain [15, 17]. Notably, radiation therapy has demonstrated the capacity to reduce the dimensions of these tumors [15, 18]. Targeted therapies, such as receptor tyrosine kinase inhibitors and histone deacetylase inhibitors, have shown promising results in some cases; however, most of these interventions have only been explored within the context of clinical trials [19].

A comprehensive analysis of the reported cases (*n* = 27), including our own case as shown in Table 2, revealed several key findings regarding clear cell sarcoma of soft tissues (CCSST). The average patient age was 31.15 ± 14.33 years, and females comprised a substantial majority (70%) of the patient population. Notably, the extremities emerged as the most common site of tumor presentation, highlighting the anatomical predilection of CCSST.

**Table 2:** Clinical and pathological characteristics of reported clear cell sarcoma of soft tissues

Case	Age (years)	Sex	Location	S100	HMB-45	Melan A	FISH
1 [6]	48	M	Neck	−	+	+	N/A
2 [8]	32	M	Penis	+	+	+	EWST/ATF1
3 [23]	31	F	Back	+	+	+	EWSR/ATF1
4 [23]	7	M	Foot	+	+	−	EWSR/ATF1
5 [23]	13	F	Arm	+	+	N/A	EWST/ATF1
6 [23]	12	F	Sole	N/A	+	+	EWST/ATF1
7 [23]	19	F	Foot	+	+	+	EWST/ATF1
8 [23]	50	F	Abdomen	+	N/A	+	EWST/ATF1
9 [23]	15	M	Sole	+	+	N/A	EWST/ATF1
10 [23]	61	F	Leg	+	+	+	EWST/ATF1
11 [23]	74	F	Arm	+	+	−	EWST/ATF1
12 [23]	65	F	Palm	+	N/A	N/A	EWST/CREB1
13 [23]	25	F	Leg	+	N/A	N/A	EWST/ATF1
14 [23]	50	F	Back	+	+	+	EWST/ATF1
15 [24]	60	M	Thigh	+	−	−	EWST/ATF1
16 [24]	12	M	Foot	+	−	+	EWST/ATF1
17 [24]	29	F	Foot	+	+	+	EWST/ATF1
18 [25]	12	M	Ankle	+	+	+	EWST/ATF1
19 [26]	25	F	Knee	+	+	N/A	EWST/ATF1
20 [27]	19	F	Wrist	+	N/A	N/A	EWST/ATF1
21 [28]	17	F	Buttock	+	N/A	+	EWST/ATF1
22 [29]	33	M	Scalp	+	+	+	EWST/ATF1
23 [29]	20	M	Face	+	+	+	EWST/ATF1
24 [30]	13	M	Lip	+	+	N/A	EWST/ATF1
25 [31]	43	F	Neck	+	+	−	EWST/ATF1
26 [9]	31	M	Pleura	+	+	N/A	N/A
27 [our case]	25	M	Foot	+	+	+	N/A

*M* male, *F* female, *HMB-45* human melanoma black 45, *EWSR/ATF* Ewings sarcoma/activating transcription factor, *CREB1* cAMP-response element binding protein

−: negative; +: positive; N/A: not available

Delving into the immunohistochemical profile, S100 protein positivity was observed in a significant proportion of cases (25 out of 27, representing 92.95%). HMB-45 positivity was found in 20 patients (74.07%), while Melan-A expression was present in 15 cases (55.56%). These findings underscore the valuable role of immunohistochemical markers in identifying CCSST.

Furthermore, the EWS/ATF-1 fusion transcript, a characteristic genetic alteration, was detected in 23 patients. Interestingly, only one case exhibited the EWSR/CREB1 rearrangement, highlighting the predominance of the EWS/ATF-1 fusion in this specific sarcoma subtype. These observations contribute to a deeper understanding of the underlying molecular landscape of CCSST.

Clear cell sarcoma of soft tissue is characterized by a grim prognosis, primarily attributed to its early propensity for dissemination and a high recurrence rate [14, 20]. Prognostic variables including necrosis, mitotic activity, resection margin, anatomical location, and tumor size exhibit negative correlations with poor prognosis [21]. Among these, tumor size at the time of surgery emerges as a pivotal determinant, displaying the strongest correlation with overall survival [22].

## Conclusion

Achieving a definitive differential diagnosis between malignant melanoma and clear cell sarcoma of soft tissues (CCSST) necessitates a comprehensive diagnostic approach. Despite the presence of overlapping morphological and immunohistochemical characteristics, meticulous histopathological evaluation remains imperative prior to resorting to molecular techniques. Additionally, contextual clinical factors, such as younger patient age and involvement of the extremities, serve to bolster the diagnostic inference for CCSST. Given the rarity of CCSST, accurate diagnostic determination heavily relies on collaborative efforts among clinicians, radiologists, and pathologists. This collaborative endeavor is indispensable in ensuring optimal patient outcomes through the facilitation of appropriate treatment modalities tailored to these uncommon neoplasms.

## Data Availability

The dataset of the current study is available from the corresponding author upon motivated request.

## Abbreviations

AJCC: American Joint Committee on Cancer
ATF1: Activating transcription factor 1
CK: Cytokeratin
CCMT: Clear cell myomelanocytic tumor
CCSST: Clear cell sarcoma of soft tissues
CREB1: CAMP-response element binding protein
EWSR1: Ewing sarcoma breakpoint region 1
FISH: Fluorescence *in situ* hybridization
FLI1: Friend leukemia integration 1
GCT-TS: Giant cell tumor of the tendon sheath
HMB45: Human melanoma black 45
IHC: Immunohistochemistry
MiTF: Microphthalmia-associated transcription factor
MM: Malignant melanoma
MPNST: Malignant peripheral nerve sheath tumor
MRI: Magnetic resonance imaging
NSE: Neuron specific enolase
PAS: Periodic acid Schiff
PLDM: Paraganglioma-like dermal melanocytic tumor
PVNS: Pigmented villonodular synovitis
RT-PCR: Reverse transcriptase polymerase chain reaction
SMA: Smooth muscle actin
TGCT: Tenosynovial giant cell tumor
